# Analgesic and Anti-Inflammatory Activities of Leaf Extract of *Mallotus repandus* (Willd.) Muell. Arg.

**DOI:** 10.1155/2014/539807

**Published:** 2014-12-31

**Authors:** Md. Mahadi Hasan, Nizam Uddin, Md. Rakib Hasan, A. F. M. Mahmudul Islam, Md. Monir Hossain, Akib Bin Rahman, Md. Sazzad Hossain, Ishtiaque Ahmed Chowdhury, Md. Sohel Rana

**Affiliations:** ^1^Laboratory of Natural Products Research, Department of Pharmacy, Jahangirnagar University, Savar, Dhaka 1342, Bangladesh; ^2^Department of Pharmacy, Jahangirnagar University, Savar, Dhaka 1342, Bangladesh

## Abstract

In folk medicine *Mallotus repandus* (Willd.) Muell. Arg. is used to treat muscle pain, itching, fever, rheumatic arthritis, snake bite, hepatitis, and liver cirrhosis. This study aimed to evaluate the antinociceptive as well as the anti-inflammatory activities of the methanol extract of leaf. The leaves were extracted with methanol following hot extraction and tested for the presence of phytochemical constituents. Analgesic and anti-inflammatory activities were evaluated using acetic acid induced writhing test, xylene induced ear edema, cotton pellet induced granuloma, and tail immersion methods at doses of 500, 1000, and 2000 mg/kg body weight. The presence of flavonoids, saponins, and tannins was identified in the extract. The extract exhibited considerable antinociceptive and anti-inflammatory activities against four classical models of pain. In acetic acid induced writhing, xylene induced ear edema, and cotton pellet granuloma models, the extract revealed dose dependent activity. Additionally, it increased latency time in tail immersion model. It can be concluded that *M. repandus* possesses significant antinociceptive potential. These findings suggest that this plant can be used as a potential source of new antinociceptive and anti-inflammatory candidates. The activity of methanol extract is most likely mediated through central and peripheral inhibitory mechanisms. This study justified the traditional use of leaf part of this plant.

## 1. Introduction

*Mallotus repandus* (Willd.) Muell. Arg. locally known as Gunti, Jhante, or Bon Natai in Bangladesh belongs to family Euphorbiaceae, an herbaceous plant which looks like a shrub or small tree that is widely distributed and naturally grown at Sundarban, Savar, and Sylhet regions in Bangladesh.* Mallotus* is one of the richest genera of the Euphorbiaceae family. The genus* Mallotus* consists of around 150 species widely distributed in tropical and subtropical regions in Asia (Cambodia, China, India, Laos, Malaysia, Sri Lanka, Thailand, and Vietnam). A small number of species are found in the North and East of Australia and the Pacific-Ocean Archipelago (the East of Fiji). Aerials parts, bark, heartwood, leaves, roots, seeds, stem bark, and whole plants are the parts of the* Mallotus* species to undertake any research work. A quantity of* Mallotus* species is acknowledged to have diverse natural compounds, principally terpenoids, polyphenols, and benzopyrans. Antioxidant, antiviral, antimicrobial, cytotoxicity and anti-inflammatory properties are different sorts of therapeutic properties shown by the isolated compounds from the extracts of* Mallotus* genus. Some of these properties are recognized in the presence of definite classes of natural compounds, for example, polyphenols responsible for the antiradical activity of* Mallotus metcalfianus* extracts [1] or benzopyrans responsible for the cytotoxicity of* Mallotus apelta* extracts [2, 3].

*Mallotus repandus* has been locally used in an herbal formula for the relief of muscle pain in Thailand [4]. In Taiwan the leaves of* Mallotus repandus* have been used as anti-inflammatory drugs [5]. In addition, it has been used as an insecticide to stop itching and as a therapy for fever, rheumatic arthritis, snake bite, hepatitis, and liver cirrhosis [6, 7]. Therefore, this study was undertaken to justify analgesic and anti-inflammatory potentials of the leaf part of* M. repandus* (MLM) using* in vivo* assay models.

## 2. Materials and Methods

### 2.1. Drugs, Chemicals, and Apparatus

Methanol was bought from SIGMA (Sigma-Aldrich, St. Louis, USA). Pentazocine and Diclofenac Na were obtained from Beximco Pharmaceuticals Ltd., Bangladesh. Heparin inj. was purchased from Rotexmedica, Germany. All the chemicals and reagents were of analytical grade.

### 2.2. Collection of Plant Material

Leaves of* Mallotus repandus* were collected from local area of Savar and authenticated by an expert taxonomist. A voucher specimen (number 39503) was deposited in the herbarium for future reference.

### 2.3. Preparation of Methanolic Extract

The collected plant parts of leaf were cleaned and washed well with water. The cleansed leaves were then partially dried by fan aeration and then fully dried in the oven at below 40°C for 4 days. The fully dried parts were then ground to a powdered form and stored in suitable condition for few days. The powdered plant materials of leaf (480 gm) were used for extraction by Soxhlet apparatus at elevated temperature (65°C) using petroleum ether and ethyl acetate (500 mL), respectively. After complete exhaustion the powder was then treated with 500 mL of methanol. Resulting methanol extract was filtered through fresh cotton bed. The filtrate obtained was dried at temperature of 40 ± 2°C to have gummy concentrate of the crude extract. It was kept in suitable container with proper labeling and stored in cold and dry place. The yield value for methanol extract was 12.79%.

### 2.4. Animals and Experimental Setup

Sprague-Dawley female rats of 120–140 g and Swiss albino female mice of 25–30 g were collected from Pharmacology Laboratory, Department of Pharmacy, Jahangirnagar University, and were acclimatized to normal laboratory conditions for one week prior to study and were assessed to pellet diet and water* ad libitum*. Temperature of facility was 25 ± 3 °C and light/darkness alternated 12 hours apart. The animals were divided into five groups of five animals each. The study was conducted following the approval by the Institutional Animal Ethical Committee of Jahangirnagar University, Savar, Dhaka, Bangladesh.

### 2.5. Phytochemical Screening

The methanol extract of* Mallotus repandus* leaf underwent phytochemical screening to detect the presence of potential phytochemical constituents like alkaloids, carbohydrates, flavonoids, saponins, tannins, glycosides, steroids, and terpenoids [8].

### 2.6. Acute Toxicity Study

According to the OECD guideline, mice were divided into five groups of ten animals each. Different doses (250, 500, 1000, 2000, and 4000 mg/kg) of methanol extract were administered by stomach tube. Then the animals were observed for general signs of toxicity.

### 2.7. Tail Immersion Test

The tail immersion method was also used to evaluate the central mechanism of analgesic activity. Here the painful reactions in animals were produced by thermal stimulus, that is, by dipping the tip of the tail in hot water [9]. Swiss albino mice were grouped and treated with 500, 1000, and 2000 mg/kg body weight, respectively. Here, pentazocine (10 mg/kg) is used as a reference drug as well. Control group received only vehicle. The animals were fasted for 16 hours with water* ad libitum*. After administration of standard and test drugs, the basal reaction time was measured by immersing the tail tips of the mice (last 1-2 cm) in hot water heated at temperature 55 ± 1°C. The actual flick response of mice, that is, time taken in seconds to withdraw it from hot water source, was calculated and results were compared with control group. Mice with baseline latencies of more than 10 s were eliminated from the study, a latency period of 15 s was set as the cutoff point, and the measurement was then stopped to avoid injury to mice. The latent period of the tail-flick response was determined at 0, 1, 2, 3, and 4 h after the administration of drugs.

### 2.8. Acetic Acid Induced Writhing

The method according to Koster et al. [10] was employed for this test. Five groups of mice (5 mice in each group) were pretreated with control, Diclofenac Na (100 mg/kg) and the extract (500, 1000 and 2000 mg/kg). Forty-five minutes later each mouse was injected i.p. with 0.7% acetic acid at a dose of 10 mL/kg body weight. The number of writhing responses was recorded for each animal during a subsequent 5 min period after 15 min i.p. administration of acetic acid and the mean abdominal writhing for each group was obtained.

The percentage inhibition of writhing was calculated using the following formula:

(1) %  Inhibition =1−No.  of  Writhing  Drug/StandardNo.  of  Writhing  Control×100.

### 2.9. Xylene Induced Ear Edema in Mice

The xylene induced ear edema test was performed as described by Dai et al. [11]. The tested samples including Diclofenac Na (100 mg/kg) as a positive control were given orally to the mice and plant extract was given as described before. One hour later, each animal received 20 *μ*L of xylene on the anterior and posterior surfaces of the right ear lobe. The left ear was considered as control. Mice were sacrificed one hour after xylene application and circular sections were taken, using a cork borer with a diameter of 3 mm, and weighed. The weight of edema was considered as the difference between weight of ear treated with xylene (right ear) and the weight of ear without xylene treatment (left ear).

The percentage inhibition of ear edema was calculated by the following formula:

(2) %  Inhibition =1−Weight  of  Edema  Drug/StandardWeight  of  Edema  Control×100.

### 2.10. Cotton Pellet Induced Granuloma Formation in Rat

The cotton pellet induced granuloma method was performed as described by Swingle and Shideman [12]. Sterilized cotton pellets of 40 ± 1 mg weight each were impregnated subcutaneously, one on each side of the abdomen of the animal, under ketamine anesthesia and sterile technique. Test drugs were administered orally to test animals, as described before, in a once-daily dosage regimen for 7 days; the control group received vehicle only. Diclofenac (100 mg/kg) was used as a reference drug. The rats were sacrificed on the 8th day and the cotton was removed and dried at 60°C for 24 hrs, and dry cotton weight was recorded. The weight difference of dry cotton and the cotton before implantation is considered as weight of granuloma formed.

The percentage inhibition of granuloma formation was calculated by the following formula:

(3) %  Inhibition =1−Weight  of  Granuloma  Drug/StandardWeight  of  Granuloma  Control×100.

### 2.11. Statistical Analysis

The results were expressed as the mean ± SEM (standard error of mean). The results were statistically analyzed using repeated measures analysis of variance (RM-ANOVA) and one way ANOVA followed by Dunnett's multiple comparison tests. *P* < 0.05 was considered as statistically significant. Statistical programs used were GRAPHPAD PRISM (version 6.00; GraphPad Software Inc., San Diego, CA, USA) and SIGMAPLOT (version 12.0, Systat Software Inc., San Jose, California, USA).

## 3. Results

### 3.1. Phytochemical Screening

Methanol extract of* M. repandus* leaf has been shown to possess different types of phytoconstituents including flavonoids, tannins, and saponins (Table 1).

### 3.2. Acute Toxicity Study

The extract administered up to high dose (4000 mg/kg) produced no mortality. The animals did not manifest any sign of restlessness, respiratory distress, general irritation, coma, or convulsion. Hence this extract is considered safe for mice.

### 3.3. Tail Immersion Test

*M. repandus* showed significant increase in latency time after the first hour at a dose of 2000 mg/kg as shown in Table 2. Pentazocine significantly increased (*P* < 0.05) latency time at each specific hour.

### 3.4. Acetic Acid Writhing Test

Table 3 showed the effect of the methanolic extract of* M. repandus* on acetic acid induced writhing in mice. At the dose of 1000 mg/kg and 2000 mg/kg body weight, the extract produced significant 54.39% and 63% writhing inhibition (*P* < 0.05) in test animals, respectively. The extract reduced number of writhing responses in a dose dependent manner. Diclofenac Na showed 83.83% inhibition of writhing.

### 3.5. Xylene Induced Ear Edema Test

Table 4 represents effects of MLM extracts with different doses in xylene induced ear edema model. Here MLM extract showed dose dependent % inhibition of ear edema. 2000 mg/kg dose of this extract presented highest significance in reduction in edema weight (*P* < 0.05) with maximum % inhibition (79.02) in comparison with other doses. The extract reduced weight of edema in a dose dependent manner. Standard Diclofenac Na was found to present the highest inhibition (88.81%) of ear edema.

### 3.6. Cotton Pellet Induced Granuloma Model

Table 5 presents the effects of different doses of methanol extract on cotton pellet granuloma test. MLM exhibited dose dependent inhibition of granular formation. 2000 mg/kg dose presented the highest granuloma formation inhibition (24.34%) with significant value *P* < 0.05. In this model the extract presented dose dependent inhibition of granuloma formation. Standard drug Diclofenac Na showed the highest 32.57% inhibition.

## 4. Discussion

As preliminary phytochemical screening performed, it could be suggested that the antinociceptive and anti-inflammatory effects of leaf extract of* M. repandus* may be due to its content likealkaloids, carbohydrates, glycosides, flavonoids, saponins, and tannins. The effect of extract with its various doses like 500 mg/kg, 1000 mg/kg, and 2000 mg/kg against acute inflammation was observed through using xylene induced ear edema in mice. This method is generally considered as one of the standard methods for detecting the effectiveness of anti-inflammatory agents [13]. Xylene initiates acute inflammatory response which leads to serious edematous changes and vasodilation of skin when topically applied to the surfaces of the ear of mice [14–17]. According to present findings where significant results were obtained at 1000 mg/kg and 2000 mg/kg doses of extract (Table 4), it can be easily hypothesized that the leaf extract of* M. repandus* may exhibit anti-inflammatory activity through inhibiting xylene induced inflammatory response and thus ultimately decreases vasodilation and edematous condition of the ear skin of mice. Findings revealed that leaf extract of* M. repandus* showed moderate to strong effect against acute inflammation.

The abdominal contraction response induced by acetic acid is a very sensitive process which is used to screen the peripheral analgesic effect [18] and such abdominal contraction response is supposed to involve local peritoneal receptors [19]. In mice, acetic acid has been attributed to the release of arachidonic acid, which also results in the synthesis of prostaglandin through the enzyme called cyclooxygenase [20]. Acetic acid produced an abdominal writhing response due to peripheral nociceptive sensitization by prostaglandin. This method is also related to increased levels of prostanoids in general, for example, prostaglandin E2 (PGE2) and prostaglandin F2*α* (PGF2*α*) as well as lipoxygenase products in peritoneal fluids [21–24]. Inflammation due to intraperitoneal injection of acetic acid also causes the release of endogenous substances like bradykinin, serotonin, and histamine, which stimulate the central nociceptive neurons [25, 26]. Any substance which causes the inhibition of the acetic acid induced writhing may have analgesic effect preferably through the inhibition of prostaglandin biosynthesis, which is actually known as a peripheral mechanism of pain inhibition. The significant analgesic effects were observed for methanolic extract of* M. repandus* at the doses of 1000 mg/kg and 2000 mg/kg which actually buttress the antinociceptive activity of this extract (Table 3). These results of the significant reduction in the number of acetic acid induced writhing responses strongly suggest that the extract retains peripheral analgesic activity through inhibition of local peritoneal receptors or arachidonic acid pathways, involving cyclooxygenases and/or lipoxygenases. Thus prostaglandin synthesis is remarkably decreased and at the same time approves traditional use of* M. repandus* for the relief of any inflammatory pain.

It has been reported that both stem and root extracts of* M. repandus* possessed a free radical scavenging activity, which could give a beneficial action against pathological alterations like edematous conditions [27]. They also found that stem and root extracts of* M. repandus* contained flavonoids (phenolic) and tannins (polyphenolic) like phytoconstituents. Both flavonoids and tannins possessed antioxidant activity because of their redox properties which let them act as reducing agents and quencher of singlet oxygen [28–31]. Our study also found tannins and flavonoids in leaf extract of* M. repandus*. Some studies have already claimed that flavonoids also possessed anti-inflammatory action [32–34]. Therefore, both anti-inflammatory and antioxidant effect could be supposed either as the protective action against any oxidative stress or inhibition of enzymes (e.g., cyclooxygenase) of prostaglandin pathway of inflammatory process.

The cotton pellet granuloma in mice is an excellent chronic inflammatory model that was selected to investigate chronic inflammation (the proliferative phase). Inflammatory response like extravasations, formation of granuloma, and various biochemical exudates due to cotton pellet can be readily detected through this technique [35]. Potent anti-inflammatory response was observed with methanolic extract of* M. repandus* at doses of 1000 mg/kg and 2000 mg/kg; thus, the extract produced a significant dose dependent inhibition of granuloma formation at the site of inflammation in animal model (Table 5), thereby suggestive of its activity in the transudative phase and proliferative phase of inflammation which ultimately leads to significant reduction in weight of cotton pellets in comparison with pellet control mice. Our earlier phytochemical study revealed the presence of flavonoids in methanolic extract of* M. repandus*. Some studies have already demonstrated the presence of various flavonoids like quercetin, hesperidin, luteolin responsible for significant antinociceptive, and/or anti-inflammatory activities [36–40]. Therefore, it could be easily assumed that the antinociceptive and anti-inflammatory effects of the methanolic extract of* M. repandus* may be due to the contents of flavonoids.

Thermal nociception model like tail immersion method was used to evaluate the central mechanism of analgesic activity which is known to elevate the pain threshold of mice towards heat [41]. It was found that the stem of* M. repandus* possesses the best anti-inflammatory activity [42]. But we found that the methanolic extract of* M. repandus* leafpossesses not only antinociceptive but also anti-inflammatory activity. This test is useful to identify the involvement of the opioid receptors in the action of any analgesic agents that give effect in the central mechanisms [43]. In this heat induced method, methanolic extract of* M. repandus* leaf particularly at 2000 mg/kg dose significantly prolonged the latency period (*P* < 0.05), which actually suggests the antinociceptive activity of the extract and which may significantly involve central mechanism of action through activation of the opioid receptor stimulation (Table 2). It was found that tannins had antinociceptive activity [44]. It was also showed that alkaloids also possessed the ability to inhibit pain perception [45]. Preliminary phytochemical studies confirmed the presence of both alkaloids and tannins. Further studies at molecular level also may provide lucid information of their sites and mechanism of actions.

The results and the above discussion are actually suggestive of the antinociceptive and anti-inflammatory actions for the methanolic extract of* M. repandus*. However, the mechanism of these actions still remains ambiguous, and the active chemical compounds responsible for both the antinociceptive and anti-inflammatory activities of the extract remained to be elucidated at molecular levels.

## 5. Conclusion

To finish off, it is not yet confirmed truly about how this leaf extract could have versatile therapeutic properties. There is every possibility of recommending that the bioactive compounds present in the methanolic leaf extract may be responsible for many sided effects. However, further detailed studies on the efficacy, potency, and safety profile would be essential to segregate the bioactive compounds and find out their underlying molecular mechanism of action which will guarantee their clinical worth.

## Figures and Tables

**Table 1:** Phytochemical constituents identified in methanol extract of *M. repandus *leaf (MLM).

Phytochemical	Name of the tests	Observed changes	Result
Alkaloids	Mayer's test	Creamy white precipitate	−
	Hager's test	Yellow crystalline precipitate	+
	Wagner's test	Brown or deep brown precipitate	−
	Dragendorff's test	Orange or orange-red precipitate	−
	Tannic acid test	Buff color precipitate	−
Carbohydrates	Molisch's test	A red or reddish violet ring is formed at the junction of two layers and on shaking a dark purple solution is formed	+
	Barfoed's test (general test for monosaccharides)	Red precipitate	−
	Fehling's test	A red or brick-red precipitate	−
	Benedict's test		+
	Test for combined reducing sugar	A brick-red precipitate	−
Glycosides	General test	Yellow color	−
	Bromine water test	yellow precipitate	+
	Test for glucoside	Production of brick-red precipitation (carried out with the hydrolyzed extract)	−
Flavonoids	Alkaline reagent test	Red color	+/−
	Shinoda test (magnesium hydrochloride reduction test)	Green to blue color	+
	Zinc hydrochloride reduction test	Red color after few minutes	−
Saponins	Frothing test	Formation of stable foam	+
Steroids	Libermann-Burchard's test	Greenish color	−
Tannins	Lead acetate test	A yellow or red precipitate	+
	Ferric chloride test	Blue green color	−
	Alkaline reagent test	Yellow to red precipitate	+
Terpenoids	Salkowski test	Yellow color appears at the lower layer	−

(+) = presence; (−) = absence; (+/−) = presence or absence not ascertained.

**Table 2:** Effects of various extracts of *M. repandus* on the latency time in tail immersion test.

Group	Doses (mg/kg)	Latency time(s)				
		0 h	1 h	2 h	3 h	4 h
Control	1% Tween 80 in water (10 mL/kg)	4.462 ± 0.196	4.278 ± 0.370	4.404 ± 0.243	3.984 ± 0.334	3.936 ± 0.314
Pentazocine	10	4.18 ± 0.256	8.4 ± 0.51^*^	8.294 ± 0.250^*^	8.028 ± 0.96^*^	6.834 ± 0.282^*^
MLM	500	4.720 ± 0.343	5.540 ± 0.431	4.408 ± 0.556	5.138 ± 0.186	5.704 ± 0.639
	1000	4.314 ± 0.136	5.990 ± 1.297	4.164 ± 0.468	5.058 ± 0.403	4.742 ± 0.511
	2000	4.778 ± 0.133	7.720 ± 1.136	6.250 ± 1.625^*^	7.540 ± 1.282^*^	6.080 ± 0.460^*^

Values are presented in mean ± SEM (*n* = 5).  ^*^ *P* < 0.05 was considered statistically significant when compared against control. Overall time effect is considered extremely significant with *F* (4, 80) = 5.910 and *P* < 0.05. Repeated measure ANOVA with Dunnet's multiple comparison was performed to analyze this data set.

**Table 3:** Effect of *Mallotus repandus* leaf methanol extract in acetic acid writhing test.

Group	Doses (mg/kg)	Number of writhing responses	Inhibition (%)
Control	1% Tween 80 in water (10 mL/kg)	8.8 ± 0.374	—
Diclofenac Na	100	1.4 ± 0.245^*^	83.833
MLM	500	6.0 ± 0.949	31.611
	1000	4.0 ± 1.304^*^	54.389
	2000	3.2 ± 0.917^*^	63

Values are presented as mean ± SEM (*n* = 5). One way ANOVA followed by Dunnett's multiple comparisons was performed to analyze this dataset.  ^*^ *P* < 0.05 was considered statistically significant when compared against control.

**Table 4:** Effect of MLM extracts of *M. repandus* in xylene induced ear edema test.

Group	Doses (mg/kg)	Ear weight difference (mg)	Inhibition (%)
Control	1% Tween 80 in water (10 mL/kg)	2.860 ± 0.206	—
Diclofenac Na	100	0.320 ± 0.0663^*^	88.811
MLM	500	2.840 ± 0.548	0.7
	1000	0.860 ± 0.420^*^	70
	2000	0.600 ± 0.164^*^	79.02

Values are presented as mean ± SEM (*n* = 5). One way ANOVA followed by Dunnet's multiple comparisons was performed to analyze this dataset.  ^*^ *P* < 0.05 was considered statistically significant when compared against control.

**Table 5:** Effects of different doses of MLM extract of *M. repandus* in cotton pellet induced granuloma model.

Group	Doses (mg/kg)	Granuloma wt. (mg/mg cotton)	Inhibition (%)
Control	1% Tween 80 in water (10 mL/kg)	4.765 ± 0.225	—
Diclofenac Na	100	3.213 ± 0.160^*^	32.57
MLM	500	4.451 ± 0.206	6.6
	1000	3.6474 ± 0.166^*^	23.5
	2000	3.605 ± 0.207^*^	24.34

Values are presented as mean ± SEM (*n* = 5). One way ANOVA followed by Dunnet's multiple comparisons was performed to analyze this dataset. ^*^ *P* < 0.05 was considered statistically significant when compared against control.
